# Structural and mechanistic investigations on *Salmonella typhimurium* acetate kinase (AckA): identification of a putative ligand binding pocket at the dimeric interface

**DOI:** 10.1186/1472-6807-12-24

**Published:** 2012-10-02

**Authors:** Sagar Chittori, Handanahal S Savithri, Mathur RN Murthy

**Affiliations:** 1 Molecular Biophysics Unit, Indian Institute of Science, Bangalore, Karnataka, 560012, India; 2 Department of Biochemistry, Indian Institute of Science, Bangalore, Karnataka, 560012, India; 3 Current address: Laboratory of Cellular and Molecular Neurophysiology, Porter Neuroscience Research Center, NICHD, NIH, DHHS, Bethesda, MD, 20892, USA

**Keywords:** Acetate metabolism, AckA-Pta pathway, Acetate and Sugar Kinases/Heat shock cognate (Hsc) 70/Actin (ASKHA) superfamily, Conformational rearrangement, Enzyme regulation

## Abstract

**Background:**

Bacteria such as *Escherichia coli* and *Salmonella typhimurium* can utilize acetate as the sole source of carbon and energy. Acetate kinase (AckA) and phosphotransacetylase (Pta), key enzymes of acetate utilization pathway, regulate flux of metabolites in glycolysis, gluconeogenesis, TCA cycle, glyoxylate bypass and fatty acid metabolism.

**Results:**

Here we report kinetic characterization of *S. typhimurium* AckA (*St*AckA) and structures of its unliganded (Form-I, 2.70 Å resolution) and citrate-bound (Form-II, 1.90 Å resolution) forms. The enzyme showed broad substrate specificity with *k*_*cat*_/*K*_*m*_ in the order of acetate > propionate > formate. Further, the *K*_*m*_ for acetyl-phosphate was significantly lower than for acetate and the enzyme could catalyze the reverse reaction (*i.e.* ATP synthesis) more efficiently. ATP and Mg^2+^ could be substituted by other nucleoside 5′-triphosphates (GTP, UTP and CTP) and divalent cations (Mn^2+^ and Co^2+^), respectively. Form-I *St*AckA represents the first structural report of an unliganded AckA. *St*AckA protomer consists of two domains with characteristic βββαβαβα topology of ASKHA superfamily of proteins. These domains adopt an intermediate conformation compared to that of open and closed forms of ligand-bound *Methanosarcina thermophila* AckA (*Mt*AckA). Spectroscopic and structural analyses of StAckA further suggested occurrence of inter-domain motion upon ligand-binding. Unexpectedly, Form-II *St*AckA structure showed a drastic change in the conformation of residues 230–300 compared to that of Form-I. Further investigation revealed electron density corresponding to a citrate molecule in a pocket located at the dimeric interface of Form-II *St*AckA. Interestingly, a similar dimeric interface pocket lined with largely conserved residues could be identified in Form-I *St*AckA as well as in other enzymes homologous to AckA suggesting that ligand binding at this pocket may influence the function of these enzymes*.*

**Conclusions:**

The biochemical and structural characterization of *St*AckA reported here provides insights into the biochemical specificity, overall fold, thermal stability, molecular basis of ligand binding and inter-domain motion in AckA family of enzymes. Dramatic conformational differences observed between unliganded and citrate-bound forms of *St*AckA led to identification of a putative ligand-binding pocket at the dimeric interface of *St*AckA with implications for enzymatic function.

## Background

Bacteria respond to frequent changes in their environment by activating metabolic pathways that allow conversion of available nutrients into metabolites of central pathways. Utilization of acetate as a source of carbon and energy requires its initial activation to acetyl-CoA [1-3]. Sequential activities of acetate kinase (AckA, EC 2.7.2.1) and phosphotransacetylase (Pta, EC 2.3.1.8) result in the interconversion of ATP, acetate and CoA to ADP, acetyl-CoA and orthophosphate via acetyl-phosphate [4,5]. In addition to being an important pathway for carbon flow, the AckA-Pta activity might control the cellular concentration of acetyl-CoA and acetyl-phosphate, which serve as important metabolic intermediates. Acetyl-CoA plays a central role in carbon metabolism [2,3], while acetyl-phosphate acts as a global signal that regulates the function of several proteins involved in flagella biosynthesis and assembly, biofilm development, colonic acid biosynthesis and type-I pilus assembly [6,7]. Studies on AckA from several organisms have shown that the enzyme is also important for xylose metabolism, phosphoryl transfer to enzyme-I of the phosphoenolpyruvate: glucose phosphotransferase system, periplasmic binding proteins and response regulator proteins of two-component systems [8-11].

Enzymes catalyzing transfer of phosphate group include wide-spread protein folds responsible for several indispensable cellular roles including signal transduction, molecular regulation and metabolic functions [12]. Acetate kinase catalyzes reversible transfer of a γ-phosphate from ATP to acetate in the presence of a divalent metal ion [9]. Similarly, propionate and butyrate kinases utilize propionate and butyrate, respectively, as preferred substrates for phosphoryl transfer from ATP to acceptor ligands [13,14]. These enzymes are hereafter referred to as acetokinase family of enzymes (Pfam: PF00871; SCOP: 53080). Extensive structural and functional studies have been carried out for AckA from the archean *Methanosarcina thermophila*[15,16]. An earlier study on AckA from *E. coli* suggested that in addition to its cognate substrate acetate, it could also phosphorylate analogous short-chain fatty acids (SCFAs) such as propionate and butyrate [5]. However, in another study, AckAs from *E. coli* and *S. typhimurium*, which share 98% sequence identity, were found to be inactive with respect to propionate and butyrate [9]. In the present study, we report the biochemical characterization and structures of *S. typhimurium* AckA (StAckA, NCBI accession: NP_461279.1, residues: 400, molecular mass: 43.3 kDa) in two crystal forms at 2.70 Å (apo, Form-I) and 1.90 Å (citrate-bound, Form-II) resolutions. Sequence and structure-based comparisons with homologous enzymes were carried out with the view of understanding their biochemical specificity, overall fold, stability, active site geometry, domain motion and plausible regulation.

## Methods

### Enzymatic characterization

Recombinant *St*AckA (residues: 415, molecular mass: 45.0 kDa), cloned into the pRSET C vector, was overexpressed in *E. coli* and purified using Ni-NTA affinity and gel-filtration chromatography as reported earlier [17]. Biochemical activity of *St*AckA was examined by two different methods depending on the direction of the reaction (acetyl-phosphate or ATP synthesis) [5,9]. Acetyl-phosphate synthesis was monitored by coupling the reaction to the oxidation of NADH in an enzyme-coupled reaction using pyruvate kinase (PK) and lactate dehydrogenase (LDH). The standard assay reaction mixture of 0.5 ml contained 50 mM HEPES-NaOH pH 7.5, 20 mM sodium-acetate, 1 mM ATP, 10 mM MgCl_2_, 5 mM phosphoenolpyruvate, 0.25 mM NADH, 15 units of PK and 20 units of LDH. In the reverse reaction, ATP synthesis was monitored by coupling the reaction to the reduction of NADP in an enzyme-coupled reaction using hexokinase (HK) and glucose-6-phosphate dehydrogenase (G6PD). The standard reaction mixture of 0.5 ml contained 50 mM HEPES-NaOH pH 7.5, 10 mM acetyl-phosphate, 1 mM ADP, 10 mM MgCl_2_, 5 mM glucose, 1 mM NADP, 10 units each of HK and G6PD. In both the cases, the reaction was initiated by addition of the enzyme and monitored for 10 min at 340 nm. All enzymatic experiments were performed in triplicates at 37°C. The enzyme could be stored for 15 days at 4°C without any significant loss of activity. Kinetic constants for *St*AckA were determined by fitting the initial velocity versus substrate concentration (six data points covering 0.1-10 *K*_*m*_) to the Michaelis-Menten equation using GraphPad Prism software (Table 1). Metal requirement of *St*AckA was examined using the method described by Rose *et al.*[5]. Briefly, the reaction of the product acetyl-phosphate with neutral hydroxylamine leading to the formation of acetyl-hydroxamate was followed spectrophotometrically, by estimating the formation of a colored complex (ferric-hydroxamate, λ_max_ = 540 nm) in the presence of ferric ions. Putative *St*AckA inhibitors were tested by incubation (15 min at 25°C) of the enzyme with the compound prior to kinetic measurements.

**Table 1:** **Kinetic constants of *St*AckA catalyzed reaction**

**Ligand**	***K***_***m***_**(mM)**	***V***_***max***_**(μmol min**^**-1**^ **mg**^**-1**^**)**	***k***_***cat***_**(s**^**-1**^**)**	***k***_***ca*t**_**/*K***_***m***_**(s**^**-1**^ **mM**^**-1**^**)**		
Formate	13.5 ± 0.3	1180 ± 20	880 ± 105	68 ± 15		
ATP _(forrmate)_^a^	0.085 ± 0.010	1160 ± 10	870 ± 120	10240 ± 80		
Acetate	1.2 ± 0.1	1560 ± 18	1180 ± 75	985 ± 62		
ATP_(acetate)_^a^	0.070 ± 0.005	1550 ± 10	1170 ± 85	16860 ± 50		
Propionate	11.2 ± 0.1	1250 ± 12	940 ± 85	85 ± 10		
ATP_(propionate)_^a^	0.075 ± 0.004	1230 ± 40	900 ± 135	11350 ± 50		
Acetyl-phosphate	0.28 ± 0.04	2350 ± 30	1764 ± 25	6300 ± 45		
ADP	0.100 ± 0.003	2340 ± 20	1750 ± 65	17645 ± 10		
**Nucleotide**^b^	**ATP**	**GTP**	**UTP**	**CTP**		
*K*_*m*_ (mM)	0.070 ± 0.005	0.078 ± 0.027	0.0960 ± 0.058	0.088 ± 0.042		
*V*_*max*_ (μmol min^-1^ mg^-1^)	1560 ± 18	1475 ± 25	1185 ± 22	950 ± 30		
**Metal**^c^	**Mg**^**2+**^	**Mn**^**2+**^	**Co**^**2+**^	**Ni**^**2+**^		
[M] ^d^	1.0 ± 0.2	1.10 ± 0.6	2.30 ± 0.3	3.50 ± 1.0		
*V*_*max*_ (μmol min^-1^ mg^-1^)	1560 ± 18	1590 ± 30	250 ± 25	70 ± 40		
**Inhibition**	**Citrate**	**Succinate**	**α-KG**	**2,4-DAB**	**2-KB**	**Malate**
[I] (mM) ^e^	63 ± 15	75 ± 20	7 ± 1.8	5 ± 2.2	3 ± 1.3	1 ± 0.5
*V*_*app*_ (μmol min^-1^ mg^-1^) ^f^	1400 ± 80	1415 ± 65	900 ± 70	650 ± 85	340 ± 50	175 ± 95

Values reported are mean ± standard deviation from three independent experiments.

^a^ Formate, acetate and propionate are shown in parenthesis to indicate that the kinetic constants for ATP were determined in their presence.

^b^ Assays were performed with acetate and Mg^2+^.

^c^ Assays were performed with acetate and ATP.

^d^ Value of [M] represents ratio of the metal ion *w.r.t.* ATP, required for the maximum activity.

^e^ Value of the [I] represents concentration of the inhibitor required to reduce the catalytic activity of *St*AckA by 50% at the saturating concentration of cognate ligand.

^f^*V*_*app*_ was measured at equimolar concentrations of the substrate and inhibitor.

### Spectroscopic studies

Circular dichroism (CD) spectra of *St*AckA in the presence of various ligands were recorded using a JASCO J715 spectropolarimeter. The protein concentration was kept at 0.5 mg ml^-1^ in 5 mM HEPES-NaOH pH 7.5 and 20 mM NaCl in a cell with a path length of 0.1 mm. Thermal denaturation was monitored by recording the CD profile at a suitable wavelength from 20 to 95°C with a rate of 1°C min^-1^ rise in temperature in a peltier controlled cell holder (JASCO). To study the effect of ligand binding on thermal stability, protein samples were preincubated for 1 h with the selected ligand (at a concentration close to their *K*_*m*_ values) prior to denaturation studies using CD.

The intrinsic fluorescence of AckA was examined using a Hitachi F-200 fluorimeter and a 1 cm path length cuvette with ~1 μM enzyme in a total volume of 250 μl buffer containing 50 mM HEPES-NaOH pH 7.5 and 200 mM NaCl. Enzyme was titrated with the selected ligand (at a concentration close to their *K*_*m*_ values) and the fluorescence spectra were measured after an incubation time of 10 min.

### Structure refinement and validation: *ab-initio* model building of residues 230–300 in Form-II *St*AckA

Crystallization, X-ray diffraction data collection and initial phase determination of both the *St*AckA crystal forms have been reported earlier [17]. Interestingly, residues 230–300 (hereafter referred to as the “variable segment”) of Form-II were found to be in a conformation completely different from that of Form-I. These residues were modeled *ab-initio* using COOT [18]*.* Briefly, modeling of the variable segment was initiated from both ends of the variable segment. This allowed construction of residues 230–237 and 295–300 in the A-subunit and 230–264 and 277–300 in the B-subunit of Form-II *St*AckA. Interestingly, unlike Form-I, Cys286 of the B-subunit was found to make an intra-subunit disulfide bond with Cys36. Inspection of the electron density map at Cys36 of A-subunit suggested that a similar disulfide bond is formed in this subunit also. Residues 279–288 of A-subunit could be built around the disulfide bond. Subsequent refinement of the model using REFMAC5 [19] led to further improvement of the electron density map which allowed tracking of the main-chain corresponding to residues 245–246, 277–278 and 289–294 of the A-subunit. Residues 238–244 and 271–276 of A-subunit and residues 265–276 of B-subunit could not be built and are probably disordered. The disordered segments mostly belong to loop regions modeled in Form-I *St*AckA. Higher B-factors for the variable segment when compared to the rest of the polypeptide reflect the inherent flexibility of this region (data not shown). In the final structure, significant electron density is observed for the modeled residues of the variable segment (Additional file 1: Figure S1). These residues of the variable segment are also well restrained to standard bond distances and angles with 91.5 and 8.5% occupying the most favored and additionally allowed regions, respectively, of the Ramachandran plot. Details of structure refinement and validation statistics for both the crystal forms of *St*AckA are summarized in Table 2.

**Table 2:** Structure refinement and validation statistics

**Parameter**	**Form-I (unliganded)**	**Form-II (citrate-bound)**
Resolution range (Å) ^a^	50.0-2.70 (2.80-2.70)	50.0-1.90 (1.97-1.90)
No. of atoms		
Protein / Water / EDO / CIT ^b^	11748 / 204 / 16 / -	5838 / 442 / 16 / 13
R-factors (%) ^c^		
R_work_ / R_free_	22.1 / 28.3	18.9 / 22.4
Correlation coefficient (%) ^d^	85.4	94.1
Wilson B-factor (Å^2^)	53.4	41.9
Average B-factor (Å^2^)		
Protein / Water / EDO / CIT	44.7 / 10.4 / 45.8 / -	36.1 / 44.2 / 56.4 / 33.9
RMS deviation		
Bond length (Å)	0.006	0.014
Bond angle (°)	0.977	1.414
Dihedral angle (°)	5.024	5.908
Chiral-center restraints (Å^3^)	0.066	0.098
General planes (Å)	0.003	0.006
Coordinate error: Luzzati (Å)	0.412	0.237
Residues in Ramachandran map (% / number)		
Most favoured region	89.1 / 1,228	90.9 / 610
Allowed region	10.9 / 150	8.8 / 59
Generously allowed region	0 / 0	0.3 / 2
Disallowed region	0 / 0	0 / 0

Refinement and validation statistics are from REFMAC5 [19] and PROCHECK [37].

^a^ Values for the highest resolution shell are given in parentheses.

^b^ EDO and CIT refer to ethylene glycol and citrate, respectively.

^c^ R_work_ = $\Sigma \left(\left|{\text{F}}_{\text{obs}}\right|-|{\text{F}}_{\text{calc}}|\right)/\Sigma \left|{\text{F}}_{\text{obs}}\right|$; R_free_ was calculated similarly by using 5% of the reflections that were excluded from the refinement.

^d^ Correlation coefficient = $\sum \left({\text{F}}_{\text{o}}{\text{F}}_{\text{c}}-<{\text{F}}_{\text{o}}><{\text{F}}_{\text{c}}>\right)/{\left(\sum \left({{\text{F}}^{2}}_{\text{o}}-<{\text{F}}_{\text{o}}{>}^{2}\right)\left(\sum {{\text{F}}^{2}}_{\text{c}}-<{\text{F}}_{\text{c}}{>}^{2}\right)\right]}^{1/2}$.

### Sequence and structural analyses

Sequence homologs of *St*AckA were identified in the Swiss-Prot database [20]. DALI [21] server was used for the identification of homologous structures. Multiple sequence alignment was achieved using ClustalW [22] and a graphical representation of the alignment as well as superposition of the secondary structures was obtained using ESPript [23]. The Protein Interfaces, Surfaces and Assemblies (PISA) [24] and Protorp [25] servers were used for the analysis of dimeric interfaces. HingeProt [26] and ElNeMo [27] servers were used to analyze plausible conformational dynamics. Computed Atlas of Surface Topography of proteins (CASTp) [28] server was used for locating potential ligand-binding cavities. Ligands were modeled at the active site of *St*AckA by comparison with the ligand bound structure of *Mt*AckA (PDB:1TUY) [16], and energy minimized using the CNS program [29]. Molecular figures were created with PyMOL (http://www.pymol.org/).

### Accession codes

The atomic coordinates and structure factors for the apo (Form-I, PDB:3SLC) and citrate-bound (Form-II, PDB:3SK3) forms of StAckA have been deposited with RCSB.

## Results

### Acetokinase family of enzymes in prokaryotes

**Figure 1:**
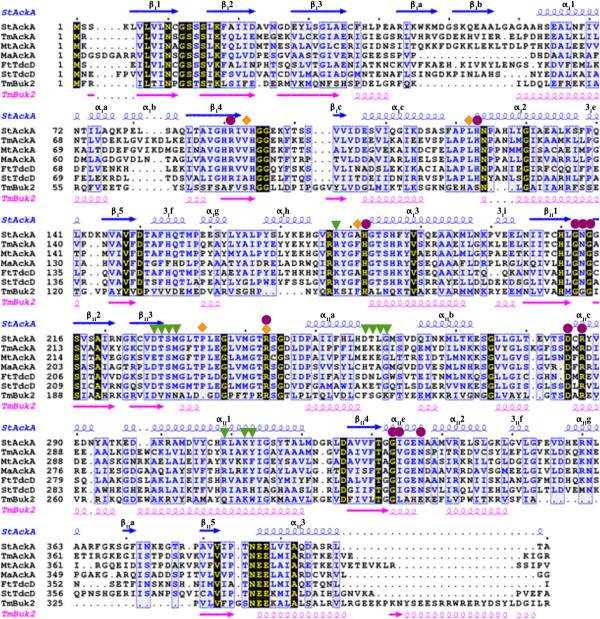
**Sequence analysis of acetokinase family of enzymes. ** Multiple sequence alignment of *St *AckA with known structures belonging to acetokinase family. Sequence code: *St*AckA, *S. typhimurium* AckA; *Tm*AckA, *Thermotoga maritima* AckA; *Mt*AckA, *Methanosarcina thermophila* AckA; *Ma*AckA, *Mycobacterium avium* AckA; *Ft*TdcD, *Francisella tularensis* putative acetate/propionate kinase; *St*TdcD, *S. typhimurium* propionate kinase; *Tm*Buk2, *Thermotoga maritima* butyrate kinase 2*.* All sequences are numbered at the beginning of each block of aligned sequences. *St*AckA numbering is indicated by every 10 residues using a dot symbol on top of the alignment. Secondary structures of Form-I *St*AckA and *Tm*Buk2 (PDB:1SAZ) aligned onto their respective sequences are also shown (refer Figure 3A for secondary structure labeling scheme). Colour code: strictly conserved residues are shown in yellow with black background; highly similar regions are shown in blue with grey background. Putative acetate and nucleotide binding residues are marked with orange rhombi and magenta circles, respectively. Residues interacting with citrate in Form-II *St*AckA structure are highlighted by green triangles.

Search for *St*AckA homologs yielded more than 300 non-redundant sequences belonging to the acetokinase family of enzymes that includes acetate, propionate and butyrate kinases. Acetate and propionate kinases share significant sequence identity (~40%) and both groups possess only a low level of identity with butyrate kinases (~20%, Figure 1). *Salmonella typhimurium* genome codes for three homologs of acetokinase family; namely AckA (acetate kinase), TdcD (propionate kinase) and PduW (putative acetate/propionate kinase). *St*AckA shares sequence identities of 41% and 40% with *St*TdcD and *St*PduW, respectively. Based on sequence analysis, an enzyme corresponding to PduW of *S. typhimurium* could not be identified in the closely related *E. coli.* In both *S. typhimurium* LT2 and *E. coli* K12, a gene corresponding to a butyrate kinase could not be detected, suggesting differences in utilization of SCFA in microorganisms.

### Enzymatic specificity of the recombinant *St*AckA

**Figure 2:**
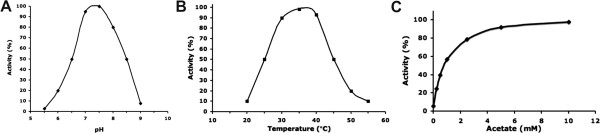
**Enzymatic characterization of *St *AckA.** Plot of enzyme activity (%) at various (**A**) pH and (**B**) Temperature values. (**C**) Profile of saturation kinetics exhibited by *St*AckA with acetate as substrate.

Earlier studies on SCFA specificity of AckAs have led to conflicting reports [5,9]. In the present study, the recombinant *St*AckA was purified to homogeneity using Ni-NTA affinity chromatography followed by gel-filtration. The preliminary enzymatic assay with acetate, ATP and Mg^2+^ as substrates showed that the *St*AckA catalyzed reaction follows Michaelis-Menten kinetics with pH and temperature optima in the range of 7.2-7.5 and 35–37°C, respectively (Figure 2). This highly purified, stable and active preparation of *St*AckA was further used to examine the kinetics of phosphoryl transfer reaction from ATP to various SCFA substrates *viz*. formate, acetate, propionate and butyrate. It was observed that *St*AckA could catalyze phosphate transfer at significant rate from ATP to formate, acetate and propionate (Table 1). However, butyrate was not found to be a suitable substrate. In addition, the presence of butyrate at equimolar concentration reduced the catalytic rate of acetate utilization by 40%, which might suggest competitive binding of butyrate at the SCFA binding site. Further, kinetic analysis showed that the *K*_*m*_ is nearly 10 times lower for acetate when compared to formate or propionate (Table 1). As maximum velocity and catalytic turnover numbers (*k*_*cat*_) were found to be similar for these substrates, the catalytic efficiency (*k*_*cat*_/*K*_*m*_) for acetate was ~10 times higher than for formate or propionate, indicating that acetate is the preferred substrate. Noticeably, the enzyme showed similar *K*_*m*_ and *k*_*cat*_ values for ATP when either formate, acetate or propionate was used as the SCFA substrate (Table 1). Further kinetic characterization revealed that *St*AckA catalyzes the reverse reaction *i.e.* phosphoryl transfer from acetyl-phosphate to ADP much more efficiently (Table 1). The *K*_*m*_ for ADP and ATP are comparable whereas the *K*_*m*_ for acetyl-phosphate is significantly lower compared to that for acetate.

The biochemical investigations were further extended for assessment of nucleotide analogs as phosphoryl donors in *St*AckA catalyzed reaction. These kinetic assays showed that apart from ATP and GTP, consistent with reports from Fox & Roseman [9], *St*AckA could also utilize pyrimidine bases such as UTP and CTP with comparable affinities (Table 1). Activity of *St*AckA was found to be completely abolished in the presence of EDTA (5 times Mg^2+^ concentration) or in the absence of suitable divalent ions, suggesting that divalent cations are essential for the catalysis. In addition to MgCl_2_ (*V*_*max*_ = 100%), the enzyme could be activated by MnCl_2_ (*V*_*max*_ = 100%), CoCl_2_ (*V*_*max*_ = 15%) and to a minor extent by NiCl_2_ (*V*_*max*_ = 5%) (Table 1). However, several other divalent (CaCl_2_, FeCl_2_, CuCl_2_, ZnCl_2_, CdCl_2_ and HgCl_2_) and monovalent (LiCl or CsCl) ions failed to activate the enzyme. A divalent metal-ion ratio of 1:1 with ATP appeared to be optimal while a large excess (>25 times) of Mg^2+^ relative to ATP was found to reduce the catalytic rate, which might suggest that free Mg^2+^ could inhibit the binding of catalytically competent ATP-Mg^2+^ complex to the enzyme.

### Crystal structure of Form-I *St*AckA

**Figure 3:**
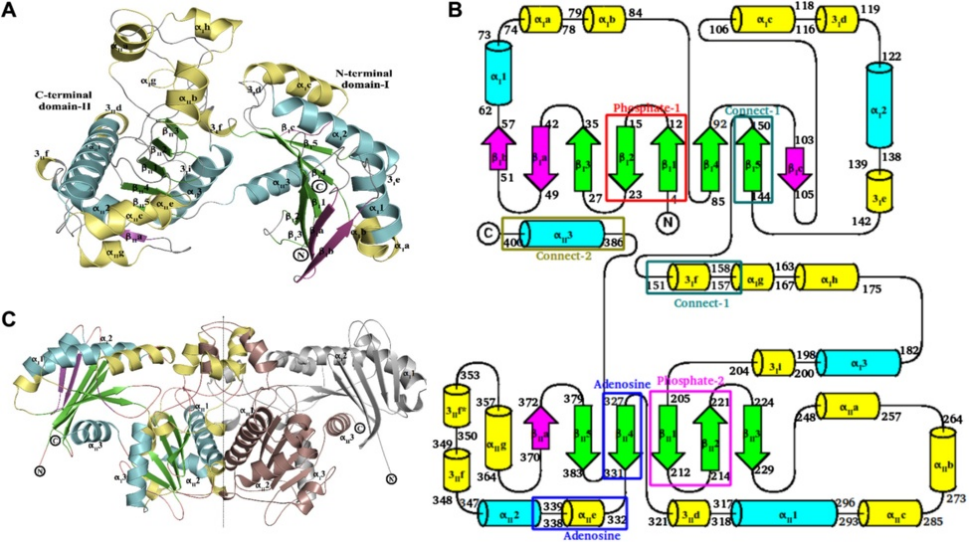
**Structure of Form-I *St *AckA. ** (**A**) Tertiary structure of *St* AckA illustrating the N- (domain-I) and C-terminal (domain-II) domains of the enzyme. The core secondary structural elements (βββαβαβα) are shown in green (strands) and cyan (helices), while insertions of subdomains between secondary structural elements are highlighted in magenta (strands) and yellow (helices). Secondary structural elements of the core are numbered α_I_1, α_I_2… for α-helices, 3_I_1, 3_I_2… for 3_10_ helices, β_I_1, β_I_2… for β-strands in domain-I and as α_II_1, α_II_2… for α-helices, 3_II_1, 3_II_2… for 3_10_ helices, β_II_1, β_II_2… for β-strands in domain-II. Insertion domains are indexed similarly and numbered alphabetically. N- and C- termini of the subunit are also marked. (**B**) Topology diagram of *St*AckA indicating the ASKHA core fold and the location of the five conserved motifs. Arrows represent β-strands while cylinders represent helices, respectively. The coloring and secondary structure labeling scheme is similar to that of Figure 3A. (**C**) Dimeric structure of *St*AckA. The approximate size of a dimeric unit is 84 x 83 x 69 Å^3^. A-subunit of the dimer is illustrated using the same colouring scheme to that of Figure 3A. Domain-I and -II of the B-subunit are shown in grey and brown, respectively. The dimer is mainly held by interactions between the C-terminal domains (domain-II) of the two subunits, while domain-I of each subunit protrudes out of the body of the dimeric enzyme. The 2-fold axis is highlighted by dotted line. Secondary structures corresponding to the core helices are labeled.

Crystal structures of two archeal acetate kinases, from *Thermotoga maritima* (PDB:2IIR, unpublished results) and *Methanosarcina thermophila*[15] and one from *Mycobacterium avium* (PDB:3P4I, unpublished results) have been determined earlier. However, structures of AckA from mesophiles such as *S. typhimurium*, or the closely related *E. coli* were not available. To obtain molecular insights into the mesophilic AckA, we determined the crystal structure of *St*AckA at 2.70 Å resolution (Form-I, Figure 3) by molecular replacement using a polyalanine model of the *Tm*AckA (PDB:2IIR, unpublished results) monomer as the search model. The asymmetric unit (ASU) of Form-I *St*AckA crystal consists of four nearly identical protomers, as indicated by the low root mean square deviations (rmsd) of corresponding C_α_ atoms (0.43-0.84 Å for >350 target pairs). *St*AckA protomer consists of 22 helices (160 residues, 41.2%), 14 β-strands (77 residues, 19.8%) and loop regions (151 residues, 38.9%; Figures 3A and B). As in the homologous archeal enzyme [15], each subunit could be further divided into N-terminal (Domain-I, residues 3–152 and 386–400) and C-terminal (Domain-II, residues 153–385) domains. The α-helices present at the carboxy-terminal ends of both the domains extend to form part of the other domain.

The four subunits of *St*AckA present in the ASU are further organized as two dimers resembling the dimeric structure of the archeal enzymes [15]. The two dimers of the asymmetric unit could be superposed with C_α_ rmsd of 0.50 Å for 706 target pairs suggesting that the organization of the protomers in the two dimers are essentially identical. The subunits of a dimer are related by nearly perfect non-crystallographic two-fold symmetries (Figure 3C). The accessible surface area (ASA) of an *St*AckA monomer was found to be 17,920 Å^2^ and upon dimerization approximately 3,061 Å^2^ (17% of total ASA) per subunit gets buried. Several segments of domain-II (α_I_g, α_I_h, α_II_a, α_II_b, α_II_1 and 3_II_f and f*) contribute to the dimeric interface. A total of approximately 81 residues (20% of total surface residues), of which 23% are polar, 53% are non-polar and 24% are charged, contribute to the interactions at the interface leading to approximately 23 hydrogen bonds and 8 salt bridges. There are also a few water-mediated hydrogen bonds between the two subunits of the dimer. This analysis indicates a stable dimeric interface.

### Active site pocket and specificity determinants

A cleft between the two domains forms the active site pocket of *St*AckA. Structural comparison with the ligand-bound *Mt*AckA structures [15,16] allowed identification of residues likely to be important for acetate (Val93, Ala181, Leu122, Pro234 and Arg243), nucleotide (Arg91, His123, His182, Gly212, Asn213, Gly214, Arg243, Asp285, Arg287, Gly333, Ile334 and Asn337) and metal-ion (Asn10, Lys17, Asp150, Glu387) binding by *St*AckA. These residues are also conserved in other members of acetokinase family (Figure 1), indicating their shared ligand binding modes and catalytic mechanisms.

**Figure 4:**
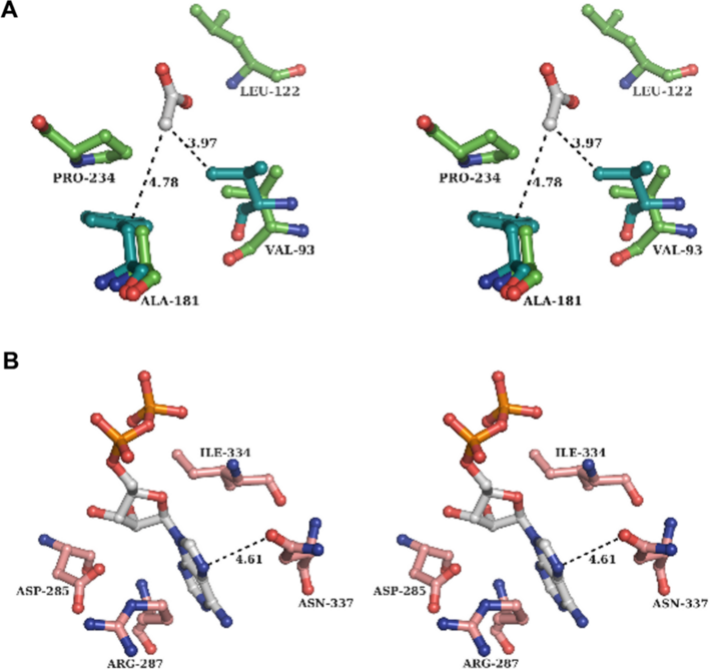
**Molecular basis for the substrate and nucleotide specificity of *St *AckA.** (**A**) Stereo diagram of SCFA binding residues (lime-green) of *St*AckA with modeled acetate (grey). For comparison, a few superposed residues (teal) of *Mt*AckA are also shown. (**B**) Stereo diagram of the putative nucleotide-binding residues (salmon-red) of *St*AckA and modeled ADP (grey). Distances shown with dashed lines are in Å.

As observed from kinetic measurements, *St*AckA could catalyze phosphoryl transfer to formate, acetate and propionate but not to butyrate (Table 1). Despite several attempts, crystals of ligand bound enzyme could not be obtained either by co-crystallization or soaking. Therefore, based on the acetate-AlF_3_-ADP bound structure of *Mt*AckA [16], formate, acetate, propionate and butyrate were modeled into the active site of the *Salmonella* enzyme. As anticipated, acetate could be accommodated with favorable interactions (Figure 4A). Although formate could also be accommodated, it made fewer interactions when compared to that of acetate while the additional methyl group of propionate (as compared to acetate) formed a short contact with Val93 of *St*AckA. This short contact could however be relieved by a small conformational adjustment of Val93 or by pointing the extra methyl-group of propionate towards Ala181, which is a semi-conserved residue and is mostly replaced by phenylalanine in acetokinases (Figures 1 and 4A). The aromatic ring of this phenylalanine bends away from the active site pocket and thus effectively presents a hydrophobic environment equivalent to that of alanine towards the SCFA binding site (Figure 4A). Similar adjustment with butyrate was not possible as the short contacts with Val93 and Ala181 could not be relieved by small conformational adjustments that would allow the reactive group of butyrate to be oriented appropriately for catalysis.

Similarly, modeling of nucleotides was carried out based on the acetate-AlF_3_-ADP bound structure of *Mt*AckA [16]. The analysis revealed that the enzyme interacts with the nucleotide base mainly by hydrophobic interactions (Figure 4B). Absence of directional ionic interactions with nucleotides could account for the broad nucleotide specificity of the enzyme. Although it is likely that AckA is primarily an ATP-dependent enzyme, the broad specificity will allow other nucleotides also to support acetate utilization.

### Ligand-induced conformational changes in *St*AckA

**Figure 5:**
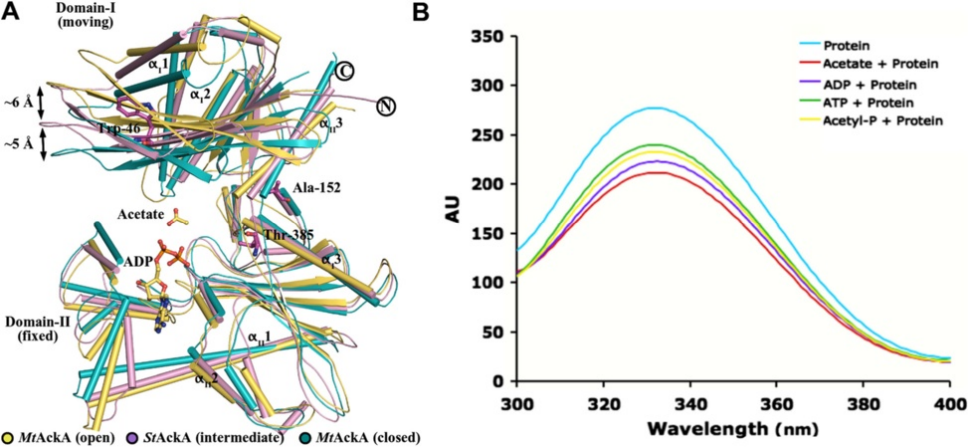
**Conformational changes induced upon ligand-binding in AckAs. (A)** Structural comparison of A-subunit of Form-I *St*AckA (apo, pink) with open (yellow) and closed (cyan) subunits of *Mt*AckA (PDB:1TUY). A large movement in domain-I (moving domain) relative to the domain-II (fixed domain) could be observed. Regions connecting the two domains are represented by the putative hinge residues (Ala152 and Thr385). N- and C-termini as well as secondary structures corresponding to the core helices are labeled. **(B)** Influence of ligand binding on the intrinsic fluorescence (excitation: 280 nm, emission: 300–400 nm) of *St*AckA.

Proteins belonging to ASKHA superfamily have been suggested to undergo inter-domain motion upon ligand binding [30-33] (Figure 5A). In the present study, ligand binding to *St*AckA was examined by monitoring the thermal stability of the enzyme using CD spectroscopy. The unliganded enzyme showed a melting temperature (T_m_) of 45°C at which 50% of the enzyme was unfolded. An increased T_m_ was observed with the addition of acetate (50°C), acetyl-phosphate (54°C), ADP (52°C) and ATP (52°C), suggesting that the protein becomes more stable upon ligand binding. Noticeably, *St*AckA contains a single tryptophan (Trp46) residue, which is located in domain-I at a large distance (20 Å between C_α_ of Trp46 and Asn213, a residue involved in binding of nucleotide phosphates) from the active site. This allows monitoring of conformational transition induced upon ligand-binding by measuring fluorescence quenching due to the change in the tryptophan environment. Intrinsic fluorescence spectra of *St*AckA recorded in the presence of acetate, acetyl-phosphate, ADP and ATP showed quenching as compared to the unliganded enzyme (Figure 5B), thus indicating that binding of these ligands is likely to result in conformational transitions that may involve inter-domain movement.

### Crystal structure of Form-II *St*AckA

**Figure 6:**
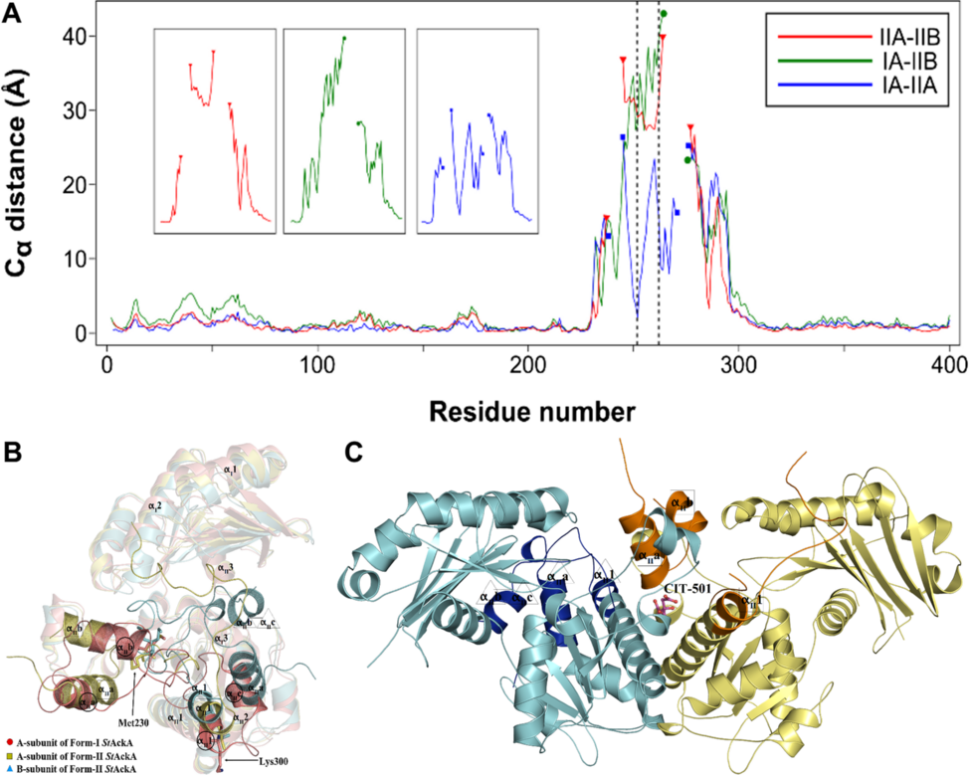
**Comparison of Form-I and -II *St *AckA.** (**A**) Plot of residual distance between sequentially equivalent C_α_ atoms against residue number obtained after pairwise superposition of A-subunit of Form-I and A- and B-subunits of Form-II *St*AckA. Labeling scheme – *e.g.* IIA-IIB corresponds to distances between equivalent C_α_ atoms after superposition of Form-II B-subunit on Form-II A-subunit. The insets show the variable segment (residues 230–300) C_α_ atom distances highlighting large conformational differences (corresponding to ~18% of the total length of the enzyme). Region corresponding to residues 251–260 (refer Additional file 1: Figure S1 for fit of the electron density) are marked by vertical dotted lines. (**B**) Structural superposition of the A-subunit of Form-I (salmon-red) with A- (yellow) and B- (cyan) subunits of the Form-II *St*AckA highlighting the structural differences of the variable segment. Secondary structures corresponding to the core helices and variable segments (labels corresponding to I-A, II-A and II-B subunits are enclosed using ○, □ and Δ shapes, respectively) are labeled. Met230 and Lys300 occur at the ends of the variable segment. (**C**) Form-II *St*AckA dimer highlighting regions of variable segment (A-subunit, yellow except for the variable segment shown in bright-orange; B-subunit, cyan with variable segment highlighted in blue). Citrate (pink, CIT-501) bound at the dimeric interface is shown in ball and stick representation. Secondary structures corresponding to the variable segments of each subunit are labeled.

Another crystal form of *St*AckA (Form-II) was obtained in the presence of citrate [17]. The structure of this form was determined at 1.90 Å resolution using a polyalanine model of the Form-I *St*AckA monomer. Although the overall polypeptide fold in these two forms is similar, residues 230–300 (variable segment) were in significantly different conformations in the two subunits of Form-II dimer and these conformations in turn were different from that observed for this segment in Form-I (Figure 6A). Superposition of A- and B-subunits of Form-II *St*AckA with the A-subunit of Form-I *St*AckA excluding the variable segment results in rmsds of 1.03 Å and 1.53 Å for 326 and 313 pairs of C_α_ atoms, respectively (Figure 6B). The variable segment is located between strand β_II_3 and helix α_II_1 (Figures 3A and B). In the A-subunit of Form-II, residues 230–237 and 247–255 are at a location close to the corresponding region of Form-I *St*AckA (Figure 6B). However, position and conformation of residues 258–270 and 277–294 are completely different from those of Form-I. The segment consisting of residues 230–263 of the B-subunit of Form-II is initially close to the corresponding segment of Form-I but progressively departs from the structure observed in Form-I towards the C-terminal end. Residues 277–287 of B-subunit are close to the active site cleft present between the domains and occupy the space where ligands are expected to bind.

The three conformationally distinct subunits (subunits of Form-I and A- and B-subunits of Form-II) also show variations in the degree of domain closure. B-subunit of Form-II represents the most closed state while A-subunit represents a conformation between those of Form-I and B-subunit of Form-II (data not shown). Despite the large conformational differences in the variable segment and inter-domain closure, structures of other segments are largely retained (Figures 6A and B) and the dimeric nature is preserved in Form-II (Figure 6C). The residues in the variable segment of Form-I are involved in inter-subunit interactions. This leads to reduction in the buried surface area (1,996 Å^2^ compared to 3,061 Å^2^ of Form-I) and ionic interactions (hydrogen bonds = 12; salt bridges =13 compared to 23 and 10, respectively, of Form-I) of the dimeric interface between the subunits (ASA of Form-II *St*AckA monomer: 17,235 Å^2^) of Form-II. As the residues of the variable segment are also involved in the formation of active site pocket, Form-II structure is not suitable for binding nucleotides and therefore may represent an inhibited state of the enzyme.

### Identification of a putative dimeric interface pocket

**Figure 7:**
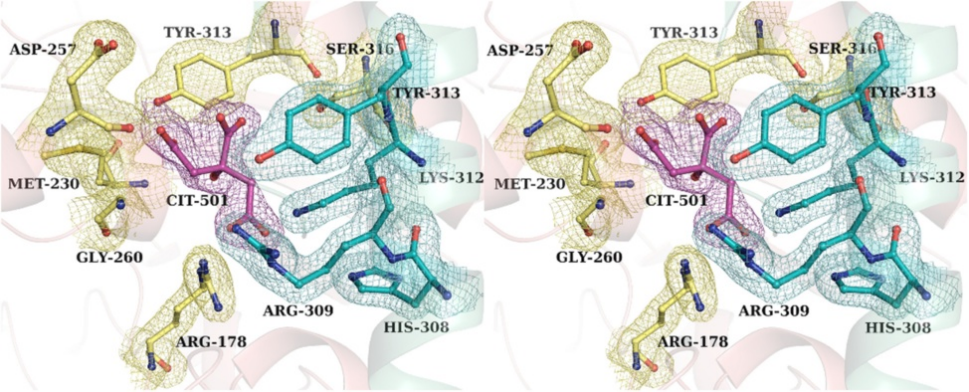
**Interactions of the citrate bound at the dimeric interface of Form-II *St *AckA.** Stereo diagram of the putative ligand-binding pocket at the dimeric interface showing bound citrate (CIT-501, magenta). Citrate anchoring residues contributed by subunit-A (pale-yellow) and subunit-B (cyan) are shown in ball and stick representation. Electron density contoured at 1σ (2F_o_-F_c_) is also shown

Examination of the difference electron density map of Form-II *St*AckA showed significant uninterpreted density located at the dimeric interface that was distinct from that of the polypeptide chain. Citrate, a component of the crystallization condition, was found to best fit the density (Figure 7) and could be refined with reasonable B-factors (33.9 Å^2^) and occupancy (0.75). Interestingly, only one citrate binds at the dimeric interface resulting in an asymmetric dimer. Citrate occupies a highly polar and charged environment. It interacts with atoms (distance cutoff: ≤5.0 Å) of residues Arg178, Asp227, Thr228, Ser229, Met230, Asp257, Thr258, Leu259, Gly260, Arg309, Lys312 and Tyr313 of the A-subunit and Arg309, Lys312 and Tyr313 of the B-subunit. Some of the residues that interact with the citrate are from the amino-terminal end of the variable segment and hence the conformational differences between the two subunits observed in the Form-II structure could be due to citrate binding.

**Figure 8:**
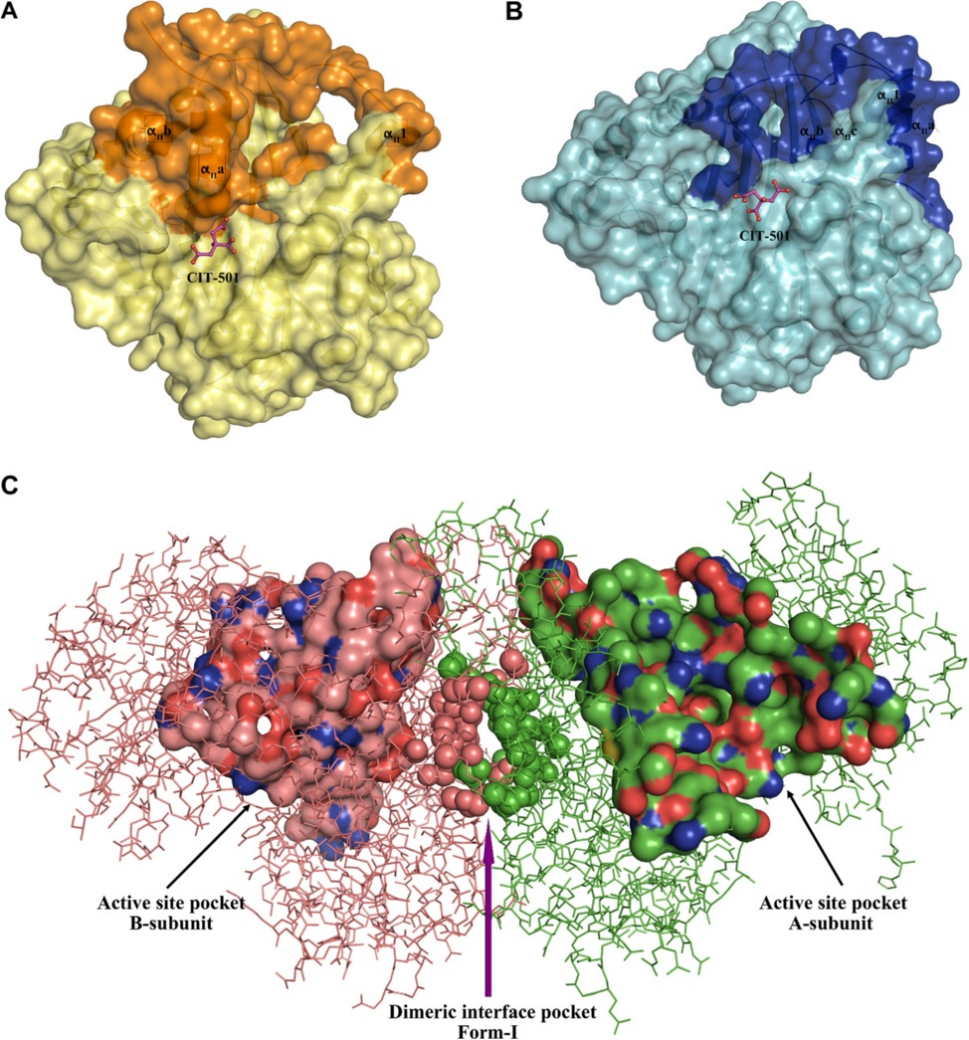
**Putative ligand binding pocket at the dimeric interface of *St *AckA.** (**A**) Representation of the A-subunit (yellow except for the variable segment shown in bright-orange) of Form-II *St*AckA with the bound citrate (CIT-501, pink, ball and stick model) indicating the location of the cavity observed at the dimeric interface. Secondary structures corresponding to the variable segments are labeled. (**B**) A similar surface representation of the B-subunit (cyan with variable segment highlighted in blue) of Form-II *St*AckA. (**C**) Dimeric interface pocket in Form-I *St*AckA. A- and B-subunits of Form-I *St*AckA are shown in green and red, respectively. Active site cavity of each subunit is shown. Dimeric interface pocket is highlighted using spheres

Inspection of Form-II *St*AckA protomer structure revealed a shallow cavity at the site of citrate binding (Figures 8A and B). In the dimeric structure, these cavities coalesce, forming a larger pocket suitable for ligand binding (Figure 8C). Intriguingly, examination of Form-I *St*AckA as well as other structurally known members of acetokinase family (Table 3) revealed a similar cavity at the dimeric interface (Figure 8C and Additional file 2: Figure S2). As many of the residues (Arg178, Asp227, Met230, Arg309 and Tyr313; *St*AckA numbering) lining the cavity are highly conserved across acetokinases (indicated by green triangle in Figure 1), the dimeric interface pocket observed for the first time in Form-II *St*AckA could be involved in binding of a putative ligand that might influence the activity of the enzyme.

**Table 3:** Structural features of the dimeric interface pocket identified in acetokinase family of enzymes

**Protein**	**PDB code**	**Identity (%)**^**a**^	**RMSD (Å**^**2**^**) / C**_**α**_**aligned**^**a**^	**Area (Å**^**2**^**)**	**Volume (Å**^**3**^**)**	**Reference**
*St*AckA-I-AB	3SLC	100	0 / 797	898	891	present study
*St*AckA-I-CD	3SLC	100	0.59 / 706	848	866	present study
*St*AckA-II-AB ^b^	3SK3	100	1.44 / 579	-	-	present study
*Mt*AckA-AB	1G99	43	1.78 / 676	985	1124	[15]
*Tm*AckA-AB	2IIR	46	1.46 / 764	961	1128	unpublished results
*Ma*AckA-AB	3P4I	41	1.74 / 708	1114	1088	unpublished results
*Ft*TdcD-AB	3KHY	45	1.45 / 732	933	1271	unpublished results
*St*TdcD-AA' ^c^	2E1Y	41	1.19 / 744	922	732	[35]
*Tm*Buk2-AF	1SAZ	16	2.37 / 604	1244	1311	unpublished results ^d^

^a^ Identity and RMSD correspond to the comparison of the query protein with *St*AckA Form-I dimer (*St*AckA-I-AB).

^b^ Due to continuity between the dimeric interface pocket and the active site cavities of A- and B-subunits in Form-II *St*AckA (Additional file 2: Figure S2C), volume of dimeric interface pocket in Form-II is not well defined.

^c^*St*TdcD dimer (A' indicates symmetry relation with A) was generated using the crystallographic 2-fold axis.

^d^ The originally published paper by Diao and Hasson (2009) has been retracted by the publisher for data ownership issues.

## Discussion

### Implications to SCFA metabolism

Acetate kinase is ubiquitously present in bacteria and archea [10] and appears to be absent in humans [34]. As AckA is involved at the junction of carbohydrate and fatty acid metabolism, it might be a suitable target for development of inhibitors [34]. We have earlier structurally and biochemically characterized *Salmonella typhimurium* propionate kinase (*St*TdcD) and demonstrated that the enzyme is specific to propionate although could utilize acetate at a 10 times lower rate [13,35]*.* In the present study, we report structural and mechanistic investigations of *St*AckA. Among the SCFAs tested, acetate was the preferred substrate although the enzyme showed significant activity with formate as well as propionate (Table 1), which is consistent with the physiological function of the enzyme in acetate metabolism. On the other hand, affinity for various nucleotides were comparable (Table 1). Modeling studies revealed that interactions with the nucleotide occur mainly through the base moiety with minor contribution from the phosphates, which is in agreement with the comparable *K*_*m*_ obtained for ADP and ATP for *St*AckA (Table 1). The observed broad specificity of *St*AckA is suggestive of its primitive origin which might have been crucial for the survival of ancestral cells that functioned with limited resources.

Interestingly, *St*AckA could catalyze the reverse reaction (ATP formation) more efficiently when compared to the forward reaction (acetyl-phosphate synthesis) (Table 1). This could serve as a major source of energy during anaerobic growth of *S. typhimurium*. The structure of ADP-AlF_3_-acetate complex of *Mt*AckA [15,16] reveals that the phosphate is likely to bind at a deeper site in the active site pocket with acetyl moiety occupying an exterior site. The phosphate is likely to form significant interactions with the protein and contribute to the higher affinity for acetyl-phosphate when compared to acetate (Table 1).

### Diversification of AckA fold

DALI search [21] revealed significant similarities of *St*AckA with over 100 non-redundant (90% sequence identity cut-off) structures deposited in the PDB. These structurally similar domains include not only kinases but also several other proteins with diverse functions such as transcriptional regulator ROK family homolog of *E. coli* MLC protein (2GUP/3.9 Å/11%), O-sialoglycoprotein endopeptidase (2IVN/4.2 Å/14%), actin (3B63/4.6 Å/11%), cell division protein FtsA (1E4G/4.1 Å/11%), plasmid segregation protein ParM (3IKY/4.2 Å/10%), diol dehydratase-reactivating factor large subunit (2D0O/4.6 Å/10%), activator of 2-hydroxyglutaryl-CoA dehydratase (1HUX/4.3 Å/15%), glycerol dehydratase reactivase α-subunit (1NBW/4.6 Å/12%), heat shock 70 kDa protein (3FZF/5.4 Å/10%), rod shape-determining protein MreB (1JCF/4.0 Å/10%), ethanolamine utilization protein EutJ (3H1Q/3.8 Å/14%) and a few other proteins of unknown function (corresponding PDB code, rmsd of Cα atoms upon superposition with *St*AckA, and sequence identity with *St*AckA are shown in parentheses). This remarkable structural and functional versatility is comparable to that of the well-known TIM-barrel proteins and is consistent with biochemical studies that suggest emergence of AckA fold early in evolution.

Although no overall sequence similarity between Acetate and Sugar Kinases/Heat shock cognate (Hsc) 70/Actin (ASKHA) superfamily could be detected, these proteins typically bind ATP and contain a duplicated core with βββαβαβα topology that harbors five highly conserved motifs *(*Adenosine, Phosphate-1, Phosphate-2, Connect-1 and Connect-2) [36] (Figure 3). The N- and C- terminal domains of ASKHA superfamily of proteins could be further divided into two subdomains. The N- and C-terminal conserved subdomains conform to the βββαβαβα topology while variable subdomains are constituted by insertions (Figures 3A and B) that may account for the functional diversity of these proteins. Among acetokinase family, a characteristic insertion corresponding to residues 150–180 of *St*AckA is present before α_I_3 of domain-I (Figures 3A and B). This segment contains a few α- and 3_10_-helices, which form part of the dimer interface. Another unique insertion (residues 354–377 of *St*AckA) in acetate and propionate kinases is found between α_II_2 and β_II_5 of domain-II. In *St*AckA, the insertion consists of an additional β-strand (β_II_a) that extends the central β-sheet, an α-helix (α_II_g) and two 3_10_ helices (3_II_f and 3_II_f*). This insertion is not found in butyrate kinase. Also, the sequence of the inserted segment is not well conserved between acetate and propionate kinases. As residues from this region interact with the base of the nucleotide, the insertion may be significant for mechanistic differences within the acetokinase family of enzymes.

### Molecular basis of thermal stability in acetokinases

Availability of several acetokinase structures from mesophilic (Form-I *St*AckA; *S. typhimurium* propionate kinase (*St*TdcD), PDB:2EIY; *Francisella tularensis* acetate/propionate kinase (*Ft*TdcD), PDB:3KHY and *Mycobacterium avium* acetate kinase (*Ma*AckA), PDB:3P4I) as well as thermophilic (*Mt*AckA, PDB:1 G99; *Thermotoga maritima* acetate kinase (*Tm*AckA), PDB:2IIR and *Tm*Buk2, PDB:1SAZ) organisms in PDB allows analysis of features that might contribute to the enhanced stability of AckA homologs found in thermophiles. Analysis of amino acid composition of these proteins revealed that the protein surfaces in thermophilic AckA homologs have a larger number of charged residues and a smaller number of polar residues when compared to mesophilic counterparts. Solvent exposed surface of thermophilic *Mt*AckA contains 23% polar, 40% non-polar and 37% charged residues compared to 30% polar, 39% non-polar and 31% charged residues, respectively, of mesophilic *St*AckA. The increase in charged residues on the surface of the thermophilic proteins results in enhanced intra-subunit ionic interactions (salt bridges: *St*AckA = 12; *Mt*AckA = 23). Also, an excess of hydrogen bonds (*St*AckA = 23; *Mt*AckA = 32) and salt-bridges (*St*AckA = 8; *Mt*AckA = 19) in the dimeric interface provides additional stability to the thermophilic enzymes. Thus, the thermophilic proteins of acetokinase family seem to achieve stability by a large number of intra- and inter-subunit ionic interactions.

### Domain motion associated with ligand binding

Superposition of Form-I *St*AckA with protomers of *Mt*AckA structure (PDB:1TUY) revealed that *St*AckA is in a conformation intermediate to that of the open and closed states of *Methanosarcina* enzyme (Figure 5A). In order to gain insights on plausible domain motion, *St*AckA and *Mt*AckA were subjected to normal mode analysis using the ELastic NEtwork MOdel (*ELNEMO)* server. The analysis revealed conformations of *St*AckA that were similar to both the open (C_α_ rmsd: 1.15 Å) and closed (C_α_ rmsd: 1.23 Å) states of *Mt*AckA subunits, and in conjunction with fluorescence studies (Figure 5B), suggests that *St*AckA is also compatible with the inter-domain movements as observed in *Mt*AckA. It is possible that the structure of Form-I *St*AckA protomer represents an intermediate state in which the active site is accessible to the solvent and ligands. The closed form may represent the active site geometry required for catalysis. The open form may facilitate expulsion of product by reducing the interactions that stabilize the bound ligands.

### Implication of dimeric interface pocket for the function of *St*AckA

The most intriguing result of the present investigations is the rearrangement and partial disorder observed in a large segment (residues 230–300) of the Form-II polypeptide (Figures 6A and B). Despite the disordered nature, modeled residues of the variable segment possess significant electron density (Additional file 1 Figure S1) and show well restrained stereochemistry [37]. Examination of the electron density revealed the presence of a citrate molecule bound at the dimeric interface (Figures 6C and 7) and led to the identification of a putative ligand binding pocket in acetokinase family of enzymes (Figure 8 and Additional file 2: Figure S2). AckA is an important enzyme that could play a key role in controlling the flux of metabolites in several pathways including glycolysis, gluconeogenesis, TCA cycle, glyoxylate bypass and fatty acid metabolism [2,3,6-8,10,11]. Examination of the thermal stability of *St*AckA incubated with 10 mM citrate showed an increase in T_m_ (55°C) by 10°C consistent with the binding of the ligand. However, at this concentration of citrate, no change in the activity of *St*AckA was observed. Therefore, it is possible that some other metabolite of these pathways bind at the putative dimeric interface pocket and regulate the enzyme activity. In this regard, some of the metabolites of TCA cycle and their analogs were tested for inhibition of *St*AckA, of which malate was found to inhibit the enzyme maximally (Table 1)*.* Further investigations on the nature of inhibition and similar studies with other members of acetokinase family enzymes might reveal the physiological significance of such a ligand-binding pocket.

## Conclusions

Biochemical studies on *St*AckA suggested that the preferred substrate of the enzyme is acetate. The enzyme showed significant catalytic rates with formate and propionate but did not utilize butyrate as a substrate. The kinetic characterization further revealed broad specificity of the enzyme with respect to nucleotides and metal ions. Structure of Form-I *St*AckA determined in the present study represents the first report of an unliganded as well as mesophilic AckA. Biophysical studies of *St*AckA and comparison with other AckA homologs provided insights into the mechanism of ligand binding, domain motion and thermal stability in AckA family of enzymes. In Form-II *St*AckA, we observed completely unexpected conformational differences in a large segment of the polypeptide between the two forms*.* This lead to the serendipitous identification of a conserved ligand binding pocket at the dimeric interface of *St*AckA and its homologs that could form a basis for further mechanistic investigations of this family of enzymes.

## Abbreviations

AckA: Acetate kinase; *St*AckA: *Salmonella typhimurium* acetate kinase; *ASKHA*: Acetate and sugar kinases/heat shock cognate (Hsc) 70/Actin.

## Competing interests

The authors declare that they have no competing interests.

## Authors’ contributions

MRN and HSS conceived and supervised the project. SC performed the experiments, analyzed the data and wrote the paper. All authors have read and approved the final manuscript.

## Supplementary Material

Additional file 1 Figure S1.Quality of the electron density corresponding to representative residues (251–260) of the variable segment. Electron density map (2F_o_-F_c_ contoured at 1σ) and the corresponding ball and stick models for residues Ile251-Gly260 of the (A) A-subunit of Form-I (salmon-red), (B) A-Subunit of Form-II (pale-yellow) and (C) B-subunit of Form-II (cyan), of *St*AckA are shown. These segments display large spatial separation in pairwise superposition of the three subunits (refer Figure 6 of the main text). Click here for file

Additional file 2 Figure S2.Putative binding pocket identified at the dimer interface in acetokinase family of enzymes. Dimeric interface pocket identified in structurally known members of acetokinase family of enzymes (refer Table 3 of the main article) are shown with cyan spheres. The two substrate binding pockets of the dimeric form are shown in green and blue spheres. Due to continuity between the dimeric interface pocket and the active site cavities of A- and B-subunits in Form-II *St*AckA, dimeric interface pocket in Form-II is not well defined. *St*TdcD dimer (A' indicates symmetry relation with A) was generated using the crystallographic 2-fold axis. PDB codes and the subunits used for the analysis are labeled. Click here for file
