# Supplementary figures and images for: Structural and mechanistic investigations on Salmonella typhimurium acetate kinase (AckA): identification of a putative ligand binding pocket at the dimeric interface
Source: BMC Struct Biol. 2012 Oct 2;12:24. doi: 10.1186/1472-6807-12-24 (PMC3475010; doi:10.1186/1472-6807-12-24)

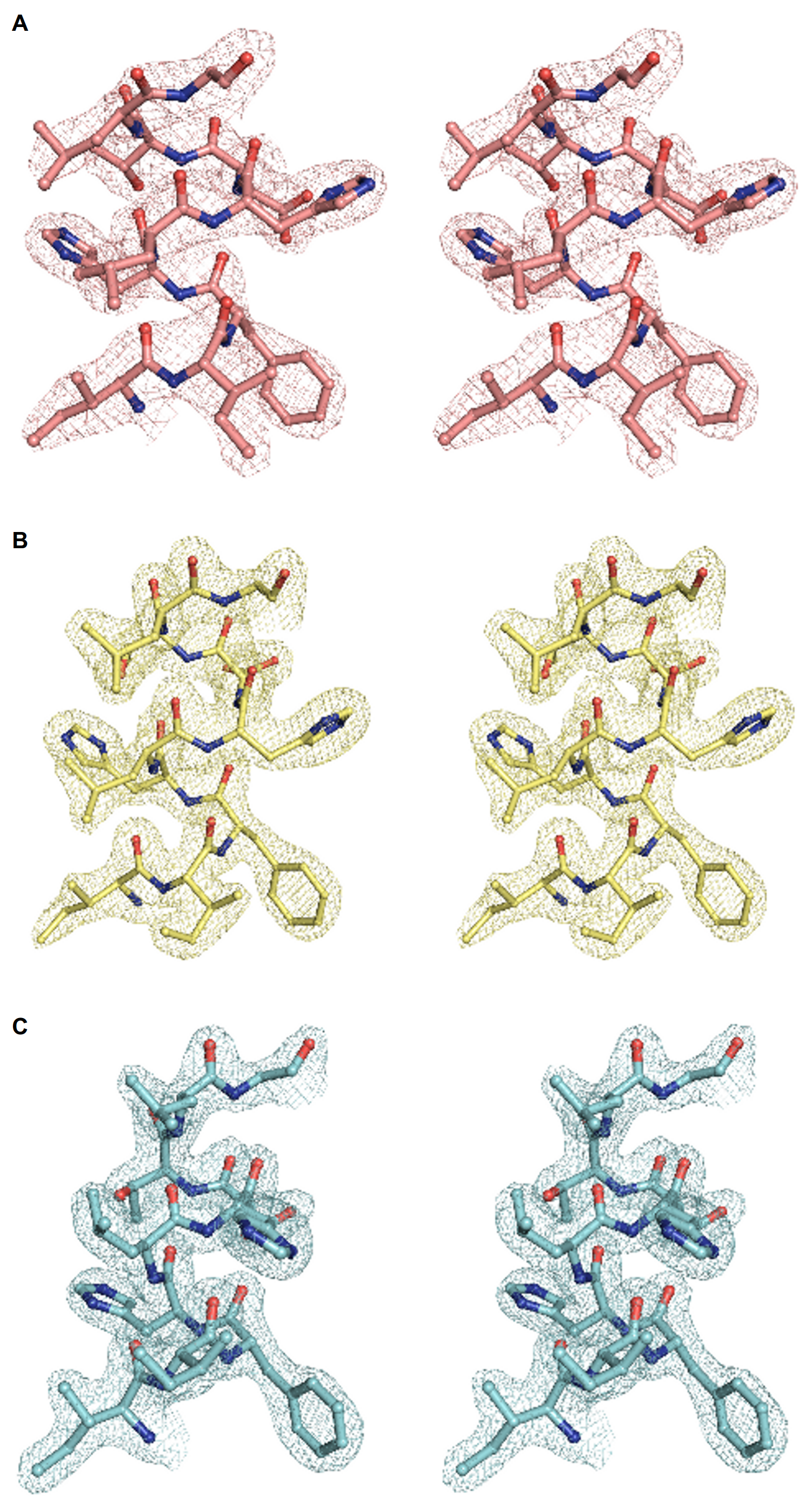

Supplement: Additional file 1 — Figure S1.Quality of the electron density corresponding to representative residues (251–260) of the variable segment. Electron density map (2Fo-Fc contoured at 1σ) and the corresponding ball and stick models for residues Ile251-Gly260 of the (A) A-subunit of Form-I (salmon-red), (B) A-Subunit of Form-II (pale-yellow) and (C) B-subunit of Form-II (cyan), of StAckA are shown. These segments display large spatial separation in pairwise superposition of the three subunits (refer Figure 6 of the main text). [file 1472-6807-12-24-S1.png]

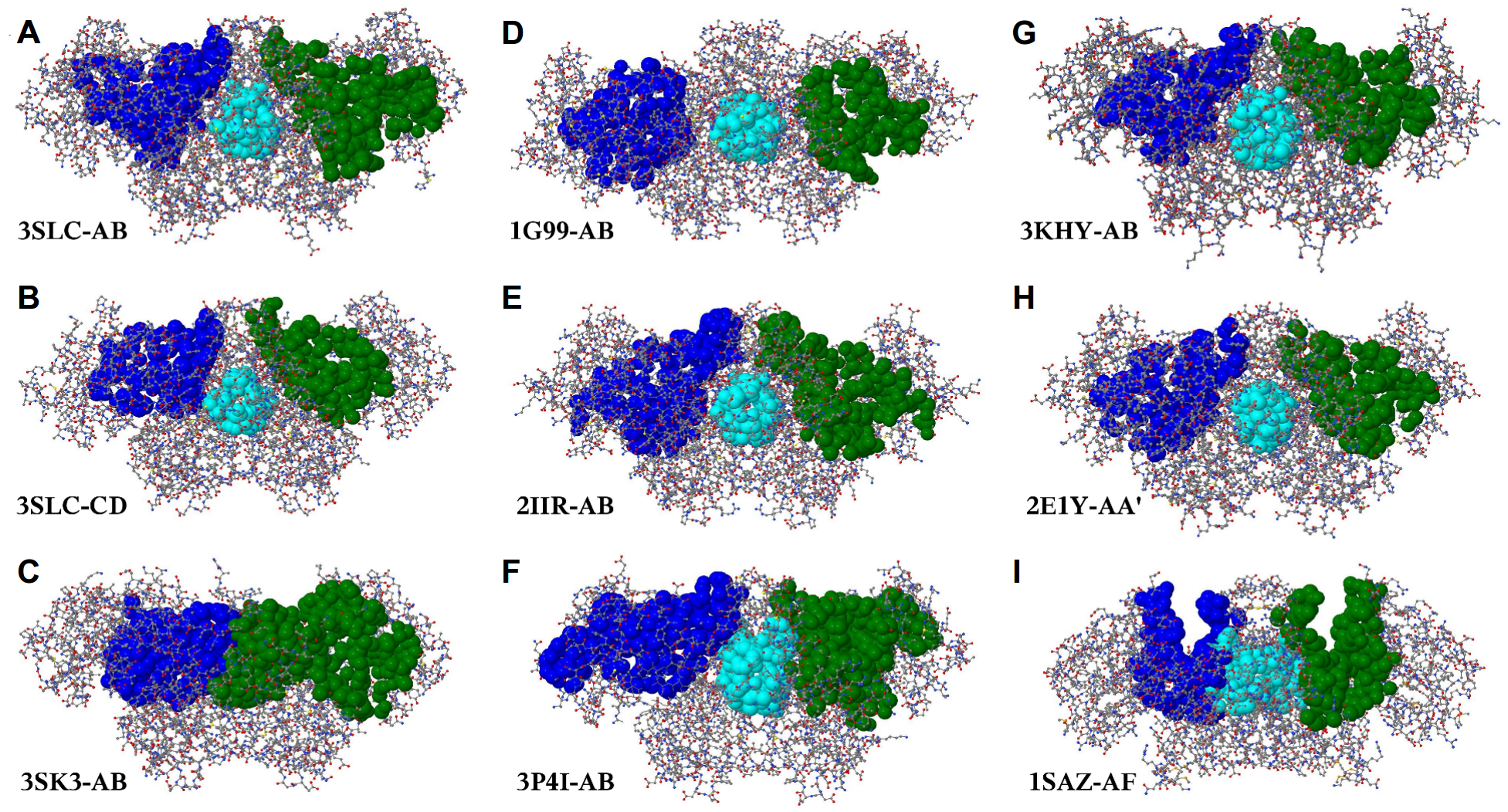

Supplement: Additional file 2 — Figure S2.Putative binding pocket identified at the dimer interface in acetokinase family of enzymes. Dimeric interface pocket identified in structurally known members of acetokinase family of enzymes (refer Table 3 of the main article) are shown with cyan spheres. The two substrate binding pockets of the dimeric form are shown in green and blue spheres. Due to continuity between the dimeric interface pocket and the active site cavities of A- and B-subunits in Form-II StAckA, dimeric interface pocket in Form-II is not well defined. StTdcD dimer (A' indicates symmetry relation with A) was generated using the crystallographic 2-fold axis. PDB codes and the subunits used for the analysis are labeled. [file 1472-6807-12-24-S2.png]
